# A study on separation of the protein structural types in amino acid sequence feature spaces

**DOI:** 10.1371/journal.pone.0226768

**Published:** 2019-12-23

**Authors:** Xiaogeng Wan, Xinying Tan

**Affiliations:** 1 College of Mathematics and Physics, Beijing University of Chemical Technology, Beijing, China; 2 The Fourth Center of PLA General Hospital, Beijing, China; National Chiao Tung University College of Biological Science and Technology, TAIWAN

## Abstract

Proteins are diverse with their sequences, structures and functions, it is important to study the relations between the sequences, structures and functions. In this paper, we conduct a study that surveying the relations between the protein sequences and their structures. In this study, we use the natural vector (NV) and the averaged property factor (APF) features to represent protein sequences into feature vectors, and use the multi-class MSE and the convex hull methods to separate proteins of different structural classes into different regions. We found that proteins from different structural classes are separable by hyper-planes and convex hulls in the natural vector feature space, where the feature vectors of different structural classes are separated into disjoint regions or convex hulls in the high dimensional feature spaces. The natural vector outperforms the averaged property factor method in identifying the structures, and the convex hull method outperforms the multi-class MSE in separating the feature points. These outcomes convince the strong connections between the protein sequences and their structures, and may imply that the amino acids composition and their sequence arrangements represented by the natural vectors have greater influences to the structures than the averaged physical property factors of the amino acids.

## Introduction

Protein is an important organics in life. It is varied with its sequence, structure, and function [1–7]. It is believed that protein functions are influenced by their structures, and the structures of proteins are influenced by their sequences [1–7]. Protein structural classification/prediction is a hot topic in bioinformatics research that particularly addresses the relations between protein sequences and their structures [8–16].

Typical protein structural classification/prediction methods are e.g. the artificial neural network methods, nearest neighbor methods, support vector machines [17]. Ding C and Dubchak I have proposed two new methods: the unique one-against-others and the all-against-all methods in protein fold classification [8]. Edler L and Grassmann J have introduced a statistical classification method including the feed forward neural networks (FFN) for the discrimination and the prediction of protein fold classes [9]. Huang C, Lin C, and Pal N have introduced three novel ideas for multiclass protein fold classification [10]. Jo T etc. have developed a deep learning network method (DN-Fold) to predict if a given query-template protein pair belongs to the same structural fold [11]. Khan M, Shahzad W and Baig A have used association rule mining technique-the ACO-AC to the problem of classifying proteins into its correct fold of the SCOP dataset [12]. Markowetz F, Edler L and Vingron M have compared the performance of support vector machines (SVMs) with neural networks methods and standard statistical classification methods such as discriminant analysis and nearest neighbor classification, where they found the SVMs provide a promising alternative to standard statistical classification and prediction methods in functional genomics [13]. Tan A, Gilbert D, and Deville Y have proposed a novel ensemble machine learning method that improves the coverage of the classifiers under the multi-class imbalanced sample sets by integrating knowledge induced from different base classifiers [14]. Wei L etc. have proposed a novel taxonomic method for protein fold prediction, called PFPA, which is featured by combining a novel feature set through an ensemble classifier [15]. Wei L and Zou Q have conducted a comprehensive review study surveying the recent computational methods, especially machine learning-based methods, in protein fold recognition [16]. Nearly all methods use protein sequence information in protein fold classification/prediction.

In this paper, we focus on the main structural classes of CATH and SCOP. The CATH database has three main structural classes, namely the mainly α structures, mainly β structures, and mixed α and β structures [17–18]. The SCOP database admits four main structural classes, namely the all α structures, all β structures, α+β structures, α/β structures [17]. In this study, we use representative protein sequence feature methods, namely the natural vector [4] and the averaged property factor [18] to present protein sequences into real-valued feature vectors. The natural vector interprets the amino acid composition and sequence arrangements of protein sequences, while the averaged property factor interprets the physical properties of amino acids for protein sequences. We use the multi-class minimum squared error (in abbreviation as the multi-class MSE) classification method [19] and convex hull classifier [20] to separate the different structural classes in feature spaces. We found that the natural vectors of different structural classes are separable by MSE hyper-planes and convex hulls, which indicates that the natural vectors of different structural classes occupy different regions in the high-dimensional feature space. The natural vector method is found to outperform the averaged property factor method in identifying the structures. This study addresses the importance of amino acid composition and sequence arrangements in identifying the structures, and the strong connections between the protein sequences and their structures.

This paper is organized as follows. In the Materials and methods section, we state the mathematical formula of the natural vector and the averaged property factor for feature extraction, and introduce the multi-class MSE and convex hull methods for feature points separation. We define the classification rates for the MSE and the convex hull methods, in order to quantify the separation of feature points. In the Results section, we describe the simulation studies on three CATH and four SCOP datasets, where we compare our feature analysis with the PseAAC [21–23] and PSSM [24–25] feature methods, and compare the classification analysis with the SVM [26] and the random forest [27–28] classification methods. In the Discussion section, the outcomes and efficiency of the structural separation, as well as the advantages and drawbacks of the feature methods and the classification methods are discussed. Finally, the conclusions of this paper are drawn in the Conclusion Section. The data of this paper are fully available and can be found in the Supporting Information Section.

## Materials and methods

In this section, we describe the natural vector (NV) and the averaged property factor (APF) methods for protein sequence feature extraction, the multi-class MSE and the convex hull methods for feature point classification, where we define the notion of classification rate that quantifies the quality of feature points separation. All these methods for feature extraction and classification are frequently used in protein classification studies [4–6, 18–20].

### Protein sequence feature extraction methods

The natural vector (NV) and the averaged property factor (APF) are two representative protein sequence extraction methods that present protein sequences from different angles.

#### The natural vector method

The natural vector method is a popular sequence feature extraction method that computes the composition and sequence arrangements of the 20 types of amino acids {A, R, N, D, C, E, Q, G, H, I, L, K, M, F, P, S, T, W, Y, V} in a protein sequence [4]. This method maps each protein sequence into a high-dimensional real-vector that uniquely represents the sequence. A protein sequence is usually composed of 20 types of amino acids {A, R, N, D, C, E, Q, G, H, I, L, K, M, F, P, S, T, W, Y, V}. Different protein sequences are varied with the frequency and arrangements of these 20 amino acids. The natural vector particularly takes the advantage of these natural parameters to interpret protein sequences.

The natural vector of a protein sequence is composed of three major parts. Firstly, the natural vector contains the quantities of the 20 amino acids in the protein sequence, which are denoted by the 20 integers *n*_*A*_, *n*_*R*_, *n*_*N*_, …, *n*_*V*_. Secondly, the natural vector contains the arithmetic mean values of the total distance for each of the 20 amino acids [4]:

$$\mu_{k}=\frac{T_{k}}{n_{k}},k=A,\;R,\;N,\;\ldots ,\;V.$$ (1)

it describes the mean distance of the k types of amino acids from the origin, *s*[*k*][*i*] is the distance from the first amino acid (regarded as origin) to the i-th amino acid k in the sequence, $T_{k}=\sum_{i=1}^{n_{k}}s[k][i]$ denotes the total distance of each of the k amino acids to the origin [4]. The third part is composed of the normalized central moments defined by [4]:

$$D_{j}^{k}=\sum_{i=1}^{n_{k}}\frac{{\left(s[k][i]-\mu_{k}\right)}^{j}}{n_{k}^{j-1}n^{j-1}},j=1,2,\ldots ,n_{k}.$$ (2)

where k represents the 20 types of amino acids in {A, R, N, D, C, E, Q, G, H, I, L, K, M, F, P, S, T, W, Y, V}, *n*_*k*_, *s*[*k*][*i*] and $\mu_{k}=\frac{T_{k}}{n_{k}}$ are defined above.

The final natural vector is a high-dimensional real vector written as [4]:

$$\langle n_{A},\mu_{A},D_{1}^{A},D_{2}^{A},\ldots ,D_{n_{A}}^{A},n_{R},\mu_{R},D_{1}^{R},D_{2}^{R},\ldots ,D_{n_{R}}^{R},\ldots ,n_{V},\mu_{V},D_{1}^{V},D_{2}^{V},\ldots ,D_{n_{V}}^{V}\rangle .$$ (3)

If a specific amino acid k does not exists, then *n*_*K*_, *μ*_*K*_, and $D_{j}^{k}$ are zeros.

Mathematically, the correspondence between a protein sequence and its natural vector is one-to-one [4]. As have been proved theoretically in [4], all the 1^st^ order central moments $D_{1}^{A},D_{1}^{R},\ldots ,D_{1}^{V}$ are zeros, so we do not need to compute them in the natural vector.

The dimension of the natural vector is quite high, which may not be efficient in computation. However, the higher central moments converge to zero very quickly [4], so the high central moments hardly make any contribution. Therefore, we can only use the first several central moments. In practice, *N* = 2 already allows us to obtain stable classified results, inclusion of higher order central moments does not change the results [4]. Therefore, we use the 60-dimensional natural vector with *N* = 2 as presented as follows [4]:

$$\langle n_{A},n_{R},\ldots ,n_{V},\mu_{A},\mu_{R},\ldots ,\mu_{V},D_{2}^{A},D_{2}^{R},\ldots ,D_{2}^{V}\rangle .$$ (4)

The 60-dimensional natural vector uniquely characterizes each protein sequence, we compute the 60 dimensional natural vectors for all protein sequences in the datasets.

#### The averaged property factor (APF) method

S. Rakovsky innovates a protein sequence feature extraction method named the average property factor (APF) method [18]. It uses the 10 physical properties of amino acids to represent protein sequences. The 10 properties of amino acids are 1. Alpha-helix/bend preference; 2. Side-chain size; 3. Extended structure preference; 4. Hydrophobicity; 5. Double-bend preference; 6. Amino acid composition; 7. Flat extended preference; 8. Occurrence in region; 9. pk; 10. Surrounding hydrophobicity. In this method, an amino acid X is represented by a 10-vector [18]

$$X=(f_{X}^{\left(1\right)},f_{X}^{\left(2\right)},\ldots ,f_{X}^{\left(10\right)}),$$ (5)

where X = A, R, N, D, C, E, Q, G, H, I, L, K, M, F, P, S, T, W, Y, V. In this expression, $f_{X}^{\left(m\right)}$ is the value of the m-th property factor of the amino acid X, m = 1, 2, …, 10, X = A, R, N, D, C, E, Q, G, H, I, L, K, M, F, P, S, T, W, Y, V. The values of the 10 property factors for each of the 20 amino acids are defined and computed by Kidera and the coworkers [29–30], which are summarized in S1 Table in the Supporting Information Section.

For every sequence S in the database, the sequence-averaged value of the m-th property factor is defined as [18]:

$${\langle f^{\left(m\right)}\rangle }_{S}=\frac{1}{N_{S}}\sum_{n=1}^{N_{S}}f_{n}^{\left(m\right)}$$ (6)

where *N*_*S*_ is the number of residues in the sequence.

The averaged property factor (APF) vector can also be computed for a predefined set Q. For example, we have a set of *N*_*Q*_ protein sequences in the set Q, each sequence corresponds to a 10 dimensional averaged property factor vector, therefore we get *N*_*Q*_ such vectors in the set Q. The averaged property factor vectors can be averaged over the *N*_*Q*_ sequences and result in one 10-dimensional APF vector:

$$V_{Q}=({\langle f^{\left(1\right)}\rangle }_{Q},{\langle f^{\left(2\right)}\rangle }_{Q},\ldots ,{\langle f^{\left(10\right)}\rangle }_{Q})$$ (7)

as the APF representation for the sequence set Q [18]. In this expression, each component

$${\langle f^{\left(m\right)}\rangle }_{Q}=\frac{1}{N_{Q}}\sum_{S\subset Q}^{\;}{\langle f^{\left(m\right)}\rangle }_{S},\;m=1,2,\ldots ,10,$$ (8)

is the average of the m-th component (property factor) over the *N*_*Q*_ sequences in the set Q.

In our study, we compute the 10 dimensional averaged property factor vector

$$V_{S}=({\langle f^{\left(1\right)}\rangle }_{S},{\langle f^{\left(2\right)}\rangle }_{S},\ldots ,{\langle f^{\left(10\right)}\rangle }_{S})$$ (9)

for every protein sequence S in the datasets.

### Structural identification methods

We first use the natural vector and the averaged property factor to compute the feature vectors for each protein sequence, then we use the multi-class MSE [19] and convex hull [20] classifiers to identify the hyper-planes and convex hull boundaries that separate the different structural classes. To quantify the separation quality, we define the notion of classification rate for the classifiers.

#### Multi-class MSE

The natural vector and the averaged property factor vectors are 60 and 10 dimensional real vectors in the high-dimensional feature spaces. The simple idea is to use the minimum squared error (MSE) classifier to compute high-dimensional hyper-planes that separate the feature points of different classes into disjoint regions.

The original MSE classifier is a bi-classification method that classifies the real-space into two disjoint regions [19]. Given the sample points of two classes, the minimum squired error (MSE) classification problem is to find the decision boundary i.e. a hyperplane that separates the points from the two classes into different regions, where the squared distances of the sample points to the decision boundary is minimized. Take the n-dimensional real-space as an example, the decision boundary can be expressed by the linear equation

$$a^{T}\left(\begin{array}{c} 1\\ x_{1}\\ \begin{array}{c} \vdots \\ x_{n} \end{array} \end{array}\right)=0,$$ (10)

where ***a*** = (*a*_0_, *a*_1_, ⋯, *a*_*n*_)^*T*^ is a weight vector. The problem in the MSE classification is to find the weight vector ***a*** that minimizes the squared errors. This can be solved by the gradient and the results can be expressed by the pseudo-inverse [19]

$$a={\left(X^{T}X\right)}^{-1}X^{T}b=X^{†}b.$$ (11)

where X is an *m* × (*n* + 1) dimensional matrix, whose rows are the augmented vectors composed of the n-dimensional sample points and the one-dimensional sign of the classes.

In practice, there are often many classes to be classified, therefore a multi-class MSE classifier is usually desired in the classifications.

Suppose there are c classes to be classified and the vector points are in d-dimensions, the multi-class MSE classification problem can be described as the problem with c linear discriminant functions [19]:

$$g_{i}(x)=a_{i}^{T}x,\;i=1,2,\ldots ,c.$$ (12)

where x is a d-dimensional column vector. For a (d-dimensional) vector point x, the multi-class MSE classifier classifies the point x into the class *ω*_*i*_ if *g*_*i*_(*x*) > *g*_*j*_(*x*), for all j ≠ i [19].

In computation, the multi-class MSE method aims to compute the d × c matrix of the weighted vectors $A=[\begin{array}{cc} a_{1} & \begin{array}{ccc} a_{2} & \cdots & a_{c} \end{array} \end{array}]$ for the c hyper-planes that separate the vector points into c disjoint regions in the d-dimensional real-space [19].

Let X be an n × d matrix of the training sample that can be written as [19]

$$X=\left[\begin{array}{c} X_{1}\\ \begin{array}{c} X_{2}\\ \vdots \\ X_{c} \end{array} \end{array}\right]$$

Each *X*_*i*_ is the sample matrix of the i-th class, whose rows are composed of the sample vectors i.e. the natural vectors of the i-th class.

Let B be n × c matrix written as [19]

$$B=\left[\begin{array}{c} B_{1}\\ \begin{array}{c} B_{2}\\ \vdots \\ B_{c} \end{array} \end{array}\right]$$

where the i-th column of each *B*_*i*_ are ones, and the other columns are zeros.

Under this notation, we get the solution of the multi-class MSE classification problem, i.e. the matrix of weighted vectors A [19]:

$$A=X^{†}B$$ (13)

which is the solution that minimizes the sum of the diagonal elements in the squared error matrix (*XA* − *B*)^*T*^ (*XA* − *B*). Here, *X*^+^ = (*X*^*T*^*X*)^−1^*X*^*T*^ denotes the pseudo inverse of X.

Once the matrix A of the weighted vectors is obtained, we judge every d-dimensional vector point x into a class *ω*_*i*_ using the criterion: *g*_*i*_(*x*) > *g*_*j*_(*x*), for all j ≠ i [19].

For the feature vectors of the structural class *i*, we use the ratio

$$R_{i}=\frac{M_{i}}{N_{i}},$$ (14)

to quantify the separation quality, which is named as the classification rate for the structural class. In this notation, *N*_*i*_ is the number of proteins (i.e. the number of feature points) in the structural class *i*, *M*_*i*_ is the number of feature points (among the *N*_*i*_ points) that are correctly classified to the structural class *i* by the multi-class MSE classifier, *i* refers to the index of the structural class, where *i* = {mainly − α, mainly − β, mixed α and β} for CATH, and *i* = {all − α, all − β, α + β, α/β} for SCOP [17]. Here, we use the protein sequence features to separate the structures, where we aim to check whether the different structural classes are separable by the sequence features.

#### Convex hull classification

Convex hull is a computational geometric concept that often used for evolutionary classification of genomes [20]. It uses convex polygon boundaries to classify vector points into convex hulls in real-spaces. For a given point set X in the (high-dimensional) real-space V, the convex hull S is the intersection of all convex sets that enclose the given point set X. The convex hull S of X can be constructed by using the convex combination of all the points {*X*_1_, *X*_2_, …, *X*_*n*_} in the set X [20].

In computation, we use the matlab toolbox function to compute the convex hull boundaries for the high-dimensional feature points, and calculate the number of points in each convex hull.

For protein feature vectors of different structural classes, we first compute the convex hull boundaries for every structural class using all feature points in this class. Then, we count the number of points that ‘exclusively’ within the convex hull of each class. We use these convex hulls to inspect whether the different structural classes are separable in terms of the feature points. To quantify the separation of the feature points, we count the number of feature points that exclusively within the convex hull of the structural class *i* and use the following ratio

$$C_{i}=\frac{A_{i}}{N_{i}},$$ (15)

to compute the classification rate for the structural class *i*, where *N*_*i*_ still denotes the number of proteins (i.e. the number of feature points) in the *i*-th structural class, and *A*_*i*_ is the number of feature points (among the *N*_*i*_ points) that exclusively enclosed in the convex hull of the *i*-th structural class. Here *i* = {mainly − α, mainly − β, mixed α and β} for CATH, and *i* = {all − α, all − β, α + β, α/β} for SCOP. In this context, the ‘exclusiveness’ refers to the feature points that solely inside or on the boundary of the *i*-th convex hull and do not appear in the other convex hulls, i.e. we seek the convex hull separation for the feature points of the different structural classes.

In practice, the computation of the convex hulls has high computational complexity, therefore the high-dimensional feature vectors are partitioned into small dimensions e.g. 10 dimensions for the convex hull classification. The classification results are listed with separate dimensions in the Results section. We use these results to examine the separation of feature points for the different structural classes.

## Results

CATH and SCOP are two protein structural classification databases, which classify proteins into different structural classes according to their secondary structures. The CATH database has three main structural classes, namely the mainly α structures, the mainly β structures, and the mixed α and β structures [17]. The SCOP database classifies proteins into four main classes, namely the all-α structures, the all-β structures, the α + β structures, the α/β structures [17]. Other structural classes are minorities. Here, we focus on the major classes of CATH and SCOP, and inspect how the structural classes are separated in terms of the different sequence features.

We use three CATH and four SCOP datasets to demonstrate the structural separation analysis. All the feature extraction analysis is compared with the PseAAC [21–23] and PSSM [24–25] analysis, and the classification analysis are compared with the SVM [26] and the random forest [27–28] analysis.

### CATH data analysis

For the CATH data, we use the three dataset examples to demonstrate the structural separation. The three examples are namely the 30 CATH groups (CATH I), the 40 CATH groups (CATH II), and the all CATH data with sequence similarity below 30% (CATH III). The 30 CATH groups (CATH I) are composed of 458 protein sequences from the three main structural classes of CATH, each class has 10 CATH groups. The 40 CATH groups (CATH II) are composed of 536 protein sequences from the three main classes of CATH, the dataset contains 14, 11, and 15 CATH groups for the mainly α, mainly β, and the mixed α & β classes respectively. The two datasets are randomly chosen in the database and have no intersection with each other. The third dataset is the set of all representative protein sequences in the PDB database with CATH classification and sequence similarity below 30%. The 30 and 40 CATH groups in the first and the second datasets are the natural CATH groups randomly selected from the CATH database. To get fair number of samples for each structural class, the CATH groups are selected randomly but to ensure that the different groups attain similar quantity level of proteins in each example. The third dataset is the entire data in the PDB database with CATH classification and sequence similarity below 30%. Details of the three datasets are shown in Table 1.

**Table 1:** Information for the CATH datasets.

Datasets		CATH I	CATH II	CATH III
**Total proteins**		458	536	8321
**Mainly α**	**Groups**	10	14	1673
	**Sequences**	157	195	
**Mainly β**	**Groups**	10	11	1772
	**Sequences**	141	145	
**Mixed α & β**	**Groups**	10	15	4876
	**Sequences**	160	196	

This table shows the group and sequence statistics for the three CATH datasets.

#### CATH I: 30 CATH groups

We compute the natural vectors (NV) and the averaged property factors (APF) for the 458 proteins of the 30 CATH groups. The protein ID and feature vectors are provided in the Supporting Information S1 and S2 Datasets. The results are compared with the PseAAC and PSSM feature analysis, and the SVM and random forest classification analysis. To inspect the effectiveness of the different features, we also test the structural separation on augmented feature spaces where the feature points are augmented vectors combined from the feature vectors of different methods. We use the multi-class MSE and the convex hulls to compute hyper-planes and convex hull boundaries that can separate the sequence features. The MSE and convex hull results are shown in Tables 2 and 3 respectively.

**Table 2:** The classification results for the 30 CATH groups by the multi-class MSE method.

Feature methods	Classification rates by structural classes (%)		
	Mainly α	Mainly β	Mixed α & β
**NV**	92.99	90.07	78.13
**APF**	83.44	53.90	60.62
**PseAAC**	91.72	67.38	71.25
**NV, APF**	93.63	90.07	83.75
**NV, PseAAC**	95.54	91.49	88.12
**APF, PseAAC**	93.63	78.02	78.02
**NV, APF, PseAAC**	96.18	89.36	90.00
**PSSM**	60.49	53.33	55.51
**Average Rates**	88.45	76.70	75.68

This table shows the MSE classification rates for the 30 CATH groups with different feature methods.

**Table 3:** The classification results for the 30 CATH groups by the convex hull method.

Feature methods		Classification rates by structural classes (%)		
		Mainly α	Mainly β	Mixed α & β
**NV**	**N1**	100	87.94	89.38
	**N2**	100	87.94	89.38
	**Mu1**	98.09	85.82	88.75
	**Mu2**	100	87.50	97.50
	**D1**	80.89	73.76	79.37
	**D2**	89.17	82.98	80.63
**APF**		79.62	86.52	89.38
**PseAAC1**		92.36	85.82	89.38
**PseAAC2**		100	85.11	89.38
**PSSM**		62.75	69.79	59.71
**Average Rates**		90.29	83.32	85.29

This table shows the convex hull classification rates for the 30 CATH groups, where the natural vectors and PseAAC vectors are partitioned into 10 dimensions. N1 refers to the first 10 dimensions of the natural vector, which are the numbers for amino acids A,R,N,D,C,Q,E,G,H,I; N2 refers to the second 10 dimensions of the natural vector, which are the numbers for amino acids L,K,M,F,P,S,T,W,Y,V. The other labels are similarly defined.

In Table 2, the average MSE classification rates for the three structural classes achieve 88.45%, 76.70% and 75.68% respectively. The results suggest that the three structural classes of CATH are separable by the MSE hyper-planes in the feature spaces, i.e. the feature points of different structural classes are separated by hyper-planes into different regions. This implies that the amino acid composition and sequence order represented by the natural vectors, and the physical properties of amino acids characterized by the averaged property factors have great importance in identifying the structures. In this table, the nature vector attains the highest classification rates than the other feature methods, and the augmented feature vectors for the combination of different features present better classification results than the individual features. Here, the combination of different features refer to the augmented feature vector containing the components of different methods, e.g. the ‘NV, APF’ in Table 2 refers to the 70 dimensional augmented feature vectors, whose first 60 dimensions are the 60 dimensional natural vectors, and the last 10 dimensions are the 10 dimensional averaged property factor vectors. The other combined features are similarly defined.

The classification rates for the convex hull method are shown in Table 3. In the convex hull classification analysis, due to the high computational complexity of the convex hulls, the high dimensional feature vectors are divided into 10 dimensions in the classification. We can see that the convex hull results have similar trends to the MSE results in the ranking of the classification rates, and the three structural classes of CATH are identifiable by all feature methods. Again, the natural vector method achieves overall higher classification rates than the other methods. In addition, the α structures have higher classification rates than the β structures.

To compare with other known classification methods, we compare our analysis with the SVM and the random forest classification analysis. The results of the SVM and the random forest are shown in Tables 4 and 5. In Table 4, the feature points of the three structural classes are well separated by the SVM method. The average classification rates (over different feature methods) for the three structural classes are 93.91%, 75.88%, and 77.13%. In the SVM classification, the natural vector method achieves the overall higher classification rates than the other methods.

**Table 4:** The classification results for the 30 CATH groups by SVM method.

Feature methods	Classification rates by structural classes (%)		
	Mainly α	Mainly β	Mixed α & β
**NV**	100	78.72	81.87
**APF**	100	60.28	71.88
**PseAAC**	100	84.40	69.37
**NV, APF**	100	79.31	77.04
**NV, PseAAC**	100	75.83	80.00
**APF, PseAAC**	100	61.54	64.00
**NV, APF, PseAAC**	99.36	94.33	85.00
**PSSM**	51.95	72.59	87.84
**Average Rates**	93.91	75.88	77.13

This table shows the classification rates for the 30 CATH groups by the SVM method.

**Table 5:** The classification results for the 30 CATH groups by random forest method.

Feature methods	Classification rates by structural classes (%)		
	Mainly α	Mainly β	Mixed α & β
**NV**	95.54	86.52	96.25
**APF**	94.90	56.74	85.00
**PseAAC**	100	92.91	95.63
**NV, APF**	95.54	87.94	96.25
**NV, PseAAC**	100	92.20	96.25
**APF, PseAAC**	100	88.65	99.38
**NV, APF, PseAAC**	100	89.36	98.12
**PSSM**	46.75	97.46	62.16
**Average Rates**	91.59	86.47	91.13

This table shows the classification rates for the 30 CATH groups by the random forest method.

Similar results are found by the random forest classification analysis. In Table 5, the random forest presents good classifications for the three structural classes. The average classification rates achieve 91.59%, 86.47%, and 91.13% for the three structural classes, the natural vector and the PseAAC methods, as well as the combined feature methods, achieve high classification rates.

From this analysis, we can see that the natural vector method outperforms the averaged property factor method in the structural separation. The former represents the amino acid compositions (the N components of the natural vector) and the positions and sequence arrangements of these amino acids (the μ and D components of the natural vector), while the latter represents the average value of the physical properties of amino acids. We may suggest that the amino acid compositions, and their sequence arrangements may have greater influence to the structures. Additionally, all classification methods present good classifications for the feature points, where the convex hull method presents better performance than the multi-class MSE in this classification analysis.

#### CATH II: 40 CATH groups

The second dataset contains 536 proteins from 40 CATH groups, which are totally different from the CATH I. The protein ID and their feature vectors are given in the Supporting Information S3 and S4 Datasets. The MSE and convex hull results are shown in Tables 6 and 7.

**Table 6:** The classification results for the 40 CATH groups by the multi-class MSE method.

Feature methods	Classification rates by structural classes (%)		
	Mainly α	Mainly β	Mixed α & β
**NV**	86.15	68.28	67.35
**APF**	82.05	43.45	60.20
**PseAAC**	85.13	46.21	70.41
**NV, APF**	88.72	66.21	70.92
**NV, PseAAC**	89.23	71.72	79.59
**APF, PseAAC**	82.05	52.41	70.92
**NV, APF, PseAAC**	88.72	69.66	80.61
**PSSM**	55.00	45.49	50.11
**Average Rates**	82.13	57.93	68.76

This table shows the multi-class MSE classification rates for the 40 CATH groups with different feature combinations.

**Table 7:** The classification results for the 40 CATH groups by the convex hull method.

Feature methods		Classification rates by structural classes (%)		
		Mainly α	Mainly β	Mixed α & β
**NV**	**N1**	97.95	91.03	93.37
	**N2**	98.46	93.10	92.35
	**Mu1**	88.21	86.90	88.78
	**Mu2**	91.79	87.59	88.78
	**D1**	94.36	88.28	89.29
	**D2**	98.46	88.28	92.35
**APF**		89.74	82.07	81.63
**PseAAC1**		84.10	86.21	84.18
**PseAAC2**		88.72	86.21	76.02
**PSSM**		69.69	79.44	72.81
**Average Rates**		90.15	86.91	85.96

This table shows the convex hull classification rates for the 40 CATH groups, where the natural vectors and the PseAAC vectors are partitioned into 10 dimensions. N1 refers to the first 10 dimensions of the natural vector, which are the numbers for the amino acids A,R,N,D,C,Q,E,G,H,I; N2 refers to the second 10 dimensions of the natural vector, which are the numbers for the amino acids L,K,M,F,P,S,T,W,Y,V. The other labels are similarly defined.

In Table 6, the three structural classes are separable by the natural vector features and the augmented vectors for the combination of different features. The average classification rates for the three structural classes are 82.13%, 57.93%, 68.76%. The mainly α structures attain the highest classification rates than the other structural classes. The natural vector method attains higher classification rates than the averaged property factor method, and the combined features tend to present improved classification rates than the features of each individual method.

The convex hull classification results are better than the multi-class MSE results. The average convex hull classification rates for the three structural classes are 90.15%, 86.91%, 85.96%. The three classes are separable by the convex hulls in terms of the feature vectors (Table 7). The natural vector method attains overall higher classification rates than the averaged property factor method in the structural identification.

The SVM classification rates are shown in Table 8. In this table, the three structural classes are well separated by using the sequence features. The SVM attains the 94.89%, 71.50%, and 79.96% average classification rates for the three structural classes. Again, the natural vector method presents higher classification rates than the averaged property factor method.

**Table 8:** The classification results for the 40 CATH groups by the SVM method.

Feature methods	Classification rates by structural classes (%)		
	Mainly α	Mainly β	Mixed α & β
**NV**	100	79.31	71.43
**APF**	100	58.62	59.69
**PseAAC**	100	57.24	75.51
**NV, APF**	100	79.31	77.04
**NV, PseAAC**	100	62.14	95.00
**APF, PseAAC**	100	53.79	88.57
**NV, APF, PseAAC**	67.37	82.86	73.33
**PSSM**	91.77	98.65	99.10
**Average Rates**	94.89	71.49	79.96

This table shows the classification rates for the 40 CATH groups by the SVM method.

The classification rates by using the random forest method is shown in Table 9. In Table 9, the average classification rates for the three structural classes are 97.84%, 79.96%, and 95.06%, and all the feature methods present good classification results. In this example, the natural vector method presents similar classification rates to the averaged property factor method for the mainly α and the mixed structures achieves, but apparently higher classification rate for the mainly β structures.

**Table 9:** The classification results for the 40 CATH groups by the random forest method.

Feature methods	Classification rates by structural classes (%)		
	Mainly α	Mainly β	Mixed α & β
**NV**	95.90	76.55	91.84
**APF**	95.38	54.48	91.84
**PseAAC**	98.46	86.21	100
**NV, APF**	96.41	80.69	93.88
**NV, PseAAC**	98.46	86.90	100
**APF, PseAAC**	99.49	83.45	100
**NV, APF, PseAAC**	99.49	86.21	99.49
**PSSM**	99.13	85.20	83.41
**Average Rates**	97.84	79.96	95.06

This table shows the classification rates for the 40 CATH groups by the random forest method.

From the analysis of this example, we can see that the natural vector method outperforms the averaged property factor method in this structural identification. All classification method present good structural separation in the feature spaces, where the convex hull method has better performance than the multi-class MSE method.

#### CATH III: CATH data with sequence similarity below 30%

In this example, we analyze all CATH data in the PDB database with sequence similarity below 30%. We downloaded the PDB data of proteins obtained by X-Ray experiments and sequence similarity below 30%. The data information is shown in Table 1. The protein ID and their feature vectors can be found in the Supporting Information S5 and S6 Datasets. We carry out the natural vector and the averaged property factor feature analysis and the multi-class MSE and convex hull classifications, the results are compared with the PseAAC and the PSSM feature analysis and the SVM and random forest classification analysis. The classification rates are shown in Tables 10–13.

**Table 10:** The classification results for the CATH data with low similarity by the multi-class MSE method.

Feature methods	Classification rates by structural classes (%)		
	Mainly α	Mainly β	Mixed α & β
**NV**	75.49	70.88	55.87
**APF**	72.80	71.78	40.07
**PseAAC**	75.37	74.77	44.93
**NV, APF**	74.42	72.52	52.38
**NV, PseAAC**	75.67	76.58	53.69
**APF, PseAAC**	75.07	73.81	46.70
**NV, APF, PseAAC**	67.90	68.96	65.67
**PSSM**	50.93	50.51	35.91
**Average Rates**	70.96	69.98	49.40

This table shows the MSE classification rates for the CATH data with sequence similarity below 30%.

**Table 11:** The classification results for the CATH data with low similarity by the convex hull method.

Feature methods		Classification rates by structural classes (%)		
		Mainly α	Mainly β	Mixed α & β
**NV**	**N1**	90.20	87.98	79.41
	**N2**	92.83	82.22	84.21
	**Mu1**	81.41	83.58	79.80
	**Mu2**	85.00	72.40	80.21
	**D1**	86.61	86.17	84.21
	**D2**	90.02	82.22	84.60
**APF**		48.95	37.25	21.33
**PseAAC1**		81.59	78.78	50.80
**PseAAC2**		90.02	79.01	58.20
**PSSM**		52.78	66.65	33.33
**Average Rates**		79.94	75.63	65.61

This table shows the convex hull classification rates for the CATH data with low similarity, where the natural vectors and PseAAC vectors are partitioned into 10 dimensions. N1 refers to the first 10 dimensions of the natural vector, which are the numbers for amino acids A,R,N,D,C,Q,E,G,H,I; N2 refers to the second 10 dimensions of the natural vector, which are the numbers for amino acids L,K,M,F,P,S,T,W,Y,V. The other labels are similarly defined.

**Table 12:** The classification results for the CATH data with low similarity by the SVM method.

Feature methods	Classification rates by structural classes (%)		
	Mainly α	Mainly β	Mixed α & β
**NV**	100	14.73	99.69
**APF**	100	39.62	73.28
**PseAAC**	100	26.30	99.57
**NV, APF**	100	47.69	98.30
**NV, PseAAC**	100	22.74	99.98
**APF, PseAAC**	100	16.25	99.10
**NV, APF, PseAAC**	57.26	22.74	100
**PSSM**	53.62	63.49	63.00
**Average Rates**	88.86	31.70	91.62

This table shows the classification rates for the CATH data with low similarity by the SVM method.

**Table 13:** The classification results for the CATH data with low similarity by the random forest method.

Feature methods	Classification rates by structural classes (%)		
	Mainly α	Mainly β	Mixed α & β
**NV**	83.56	91.20	80.66
**APF**	60.97	79.35	74.90
**PseAAC**	94.74	96.28	96.16
**NV, APF**	85.59	93.74	83.22
**NV, PseAAC**	95.28	96.95	96.29
**APF, PseAAC**	93.31	97.12	97.66
**NV, APF, PseAAC**	95.52	98.36	91.55
**PSSM**	99.40	53.44	86.67
**Average Rates**	88.55	88.31	88.39

This table shows the classification rates for the CATH data with low similarity by the random forest method.

In Table 10, the multi-class MSE method presents the average classification results of 70.96%, 69.98%, 49.40% for the three structural classes. The natural vector feature presents overall higher classification rates than the averaged property factor method. In the convex hull classification analysis (Table 11), the average classification rates for the three structural classes are 79.94%, 75.63%, 65.61%, which are overall better than the results obtained by the multi-class MSE method (Table 10). The natural vector feature well separates the three structural classes, while the averaged property factor method failed in differentiating the different classes.

The SVM and the random forest classification results are shown in Tables 12 and 13. In the SVM analysis, due to the large number of data and the high dimensions, the SVM toolbox function in matlab returns no convergence when using the entire dataset, therefore we use uniform window of W = 1500 data points and random generator to randomly select 1500 sample points for each structural class from the entire dataset, and do the SVM classifications on the randomly selected sample points. We repeat this process 10 times, where the 10 times repeats are performed independently, we compute the average classification rates for the SVM analysis. The SVM presents the average classification rates of 88.86%, 31.70%, and 91.62%. The random forest method is used on the entire dataset, it presents the average classification rates of 88.55%, 88.31%, 88.39% for the three structural classes. All structural classes are well classified by the random forest method.

### SCOP data analysis

In this section, we use four SCOP datasets to demonstrate the classification analysis. The SCOP data are randomly chosen from the four main structural classes of SCOP (all α, all β, α + β, α/β). The first dataset is a set of 24 SCOP groups (labeled by SCOP I) composed of 817 proteins with 6 groups from each structural class. The second dataset is a set of 40 SCOP groups (labeled by SCOP II) composed of 406 proteins with 10 groups from each structural class. The third dataset is a set of 48 SCOP groups (labeled by SCOP III) composed of 2509 proteins with 12 groups from each structural class. The three datasets are randomly chosen in the database and have no intersection with each other. The fourth dataset is the set of all representative protein sequences in the PDB database with SCOP classification and sequence similarity below 30%. The SCOP groups in the first three examples are the natural SCOP groups randomly selected from the SCOP database. To get fair number of samples for each structural class, the SCOP groups are selected randomly but to ensure that the different groups attain similar quantity level of proteins in each example. The fourth dataset is the entire data in the PDB database with SCOP classification and sequence similarity below 30%. Details of the SCOP data are given in Table 14.

**Table 14:** Information for the SCOP datasets.

Datasets		SCOP I	SCOP II	SCOP III	SCOP IV
**Total proteins**		817	406	2509	4836
**All α**	**Groups**	6	10	12	960
	**Sequences**	202	104	611	
**All β**	**Groups**	6	10	12	1030
	**Sequences**	205	94	568	
**α+β**	**Groups**	6	10	12	1356
	**Sequences**	213	94	651	
**α/β**	**Groups**	6	10	12	1490
	**Sequences**	197	114	679	

This table presents the group and sequence numbers for each of the four SCOP datasets.

#### SCOP I: 24 SCOP groups

This dataset contains 24 SCOP groups, we use the multi-class MSE and the convex hull methods to classify the feature points. The protein ID and their feature vectors of the 24 SCOP groups are given in the Supporting Information S7 and S8 Datasets. The results are compared with the PseAAC and the PSSM features analysis, and the SVM and random forest classification methods. The classification rates are shown in Tables 15–18. In Table 15, the four structural classes of SCOP (All α, All β, α + β, α/β) are separable by the MSE hyper-planes in the natural vector and the PseAAC feature spaces. The average classification rates for the four structural classes by the multi-class MSE method are 77.98%, 84.77%, 69.58% and 70.55%. We can see that the natural vector presents overall higher classification rates than the other methods, and nearly all combined features achieve higher classification rates than their individual features. The results for the combined features of all three methods attain the highest classification rates for the structural separation.

**Table 15:** The classification results for the 24 SCOP groups by the multi-class MSE method.

Feature methods	Classification rates by structural classes (%)			
	All α	All β	α + β	α/β
**NV**	72.77	85.85	75.12	72.59
**APF**	76.24	79.02	47.42	31.47
**PseAAC**	69.80	89.76	57.75	68.02
**NV, APF**	92.08	89.27	82.16	76.65
**NV, PseAAC**	93.07	91.22	81.69	87.82
**APF, PseAAC**	72.28	89.76	60.09	69.54
**NV, APF, PseAAC**	93.56	91.71	82.16	87.82
**PSSM**	54.04	61.54	70.22	70.51
**Average Rates**	77.98	84.77	69.58	70.55

This table shows the multi-class MSE classification rates for the 24 SCOP groups with different feature combinations.

**Table 16:** The classification results for the 24 SCOP groups by the convex hull method.

Feature methods		Classification rates by structural classes (%)			
		All α	All β	α + β	α/β
**NV**	**N1**	95.54	91.71	95.31	97.46
	**N2**	100	87.80	99.53	95.94
	**Mu1**	93.56	95.61	93.90	88.83
	**Mu2**	99.01	80.49	91.55	96.95
	**D1**	86.63	78.05	80.75	71.07
	**D2**	78.22	71.22	79.81	77.66
**APF**		97.52	31.22	84.51	88.83
**PseAAC1**		91.58	96.59	75.59	91.88
**PseAAC2**		100	79.51	72.77	90.86
**PSSM**		80.40	52.43	94.03	94.59
**Average Rates**		92.25	76.46	86.78	89.41

This table shows the convex hull classification rates for the 24 SCOP groups, where the natural vectors and the PseAAC vectors are partitioned into 10 dimensions. N1 refers to the first 10 dimensions of the natural vector, which are the numbers for amino acids A,R,N,D,C,Q,E,G,H,I; N2 refers to the second 10 dimensions of the natural vector, which are the numbers for amino acids L,K,M,F,P,S,T,W,Y,V. The other labels are similarly defined.

**Table 17:** The classification results for the 24 SCOP groups by the SVM method.

Feature methods	Classification rates by structural classes (%)			
	All α	All β	α + β	α/β
**NV**	100	89.76	64.79	86.80
**APF**	100	68.29	49.77	73.60
**PseAAC**	100	91.71	73.71	76.65
**NV, APF**	100	95.61	69.48	90.86
**NV, PseAAC**	100	96.59	76.53	89.85
**APF, PseAAC**	100	93.66	35.21	80.71
**NV, APF, PseAAC**	97.52	96.59	79.34	90.36
**PSSM**	81.12	100	95.28	100
**Average Rates**	97.33	91.53	68.01	86.10

This table shows the classification rates for the 24 SCOP groups by the SVM method.

**Table 18:** The classification results for the 24 SCOP groups by the random forest method.

Feature methods	Classification rates by structural classes (%)			
	All α	All β	α + β	α/β
**NV**	90.10	82.44	77.00	86.80
**APF**	74.75	77.56	63.38	59.90
**PseAAC**	100	99.51	100	100
**NV, APF**	92.08	82.93	78.40	88.32
**NV, PseAAC**	100	100	100	98.48
**APF, PseAAC**	100	99.51	100	100
**NV, APF, PseAAC**	98.02	95.61	96.71	96.95
**PSSM**	100	50.00	100	100
**Average Rates**	94.37	85.95	89.44	91.31

This table shows the classification rates for the 24 SCOP groups by the random forest method.

Among all structural classes, the mixed structures i.e. the α + β and α /β structures are less identifiable than the pure structures (i.e. the all α structures and the all β structures).

Table 16 presents the convex hull classification for the different features. In Table 16, the convex hull results are better than the MSE results, the four structural classes are identifiable by the natural vector. The average classification rates for the four structural classes are 92.25%, 76.46%, 86.78%, 89.41%. Among the three feature methods, the natural vector method attains higher classification rates than the averaged property factor method. This again demonstrates the importance of the amino acid composition and their sequence arrangements in identifying the structures.

Tables 17 and 18 show the classification rates by using the SVM and random forest methods. The SVM achieves the average classification rates of 97.33%, 91.53%, 68.01%, 86.10% for the four structural classes. The random forest method achieves the average classification rates of 94.37%, 85.95%, 89.44%, 91.31%. The natural vector method achieves higher classification rates than the averaged property factor method.

#### SCOP II: 40 SCOP groups

The second SCOP example is the set of 40 SCOP groups. The protein ID and feature vectors of the 40 SCOP groups are given in the Supporting Information S9 and S10 Datasets. The classification results of this example are shown in Tables 19–22. In Table 19, the four structural classes are separable in terms of the natural vector and PseAAC features. The average classification rates for the four structural classes are 71.82%, 62.86%, 60.01%, 84.54%. We can see that the natural vector method presents overall higher classification results than the other methods, and nearly all combined features have improved classification results than their individual methods. Note that the averaged property factor method attains the lowest classification rates in all structural classifications, particularly for α + β structures.

**Table 19:** The classification results for the 40 SCOP groups by the multi-class MSE method.

Feature methods	Classification rates by structural classes (%)			
	All α	All β	α + β	α/β
**NV**	66.35	71.28	75.53	91.23
**APF**	50.96	51.06	8.51	82.46
**PseAAC**	62.50	50.00	48.94	81.58
**NV, APF**	84.62	73.40	76.60	92.98
**NV, PseAAC**	89.42	74.47	79.79	89.47
**APF, PseAAC**	69.23	51.06	47.87	82.46
**NV, APF, PseAAC**	90.38	77.66	81.91	92.98
**PSSM**	61.09	53.96	60.89	63.18
**Average Rates**	71.82	62.86	60.01	84.54

This table shows the multi-class MSE classification rates for the 40 SCOP groups with different feature combinations.

**Table 20:** The classification results for the 40 SCOP groups by the convex hull method.

Feature methods		Classification rates by structural classes (%)			
		All α	All β	α+β	α/β
**NV**	**N1**	100	90.43	80.85	100
	**N2**	100	81.91	81.91	100
	**Mu1**	99.04	81.91	78.72	91.23
	**Mu2**	94.23	77.66	68.09	85.09
	**D1**	92.31	77.66	60.64	71.05
	**D2**	95.19	81.91	63.83	66.67
**APF**		82.69	89.36	80.85	88.60
**PseAAC1**		90.38	90.43	80.85	99.12
**PseAAC2**		97.12	81.91	80.85	85.09
**PSSM**		92.81	99.48	94.45	96.21
**Average Rates**		94.38	85.27	77.10	88.31

This table shows the convex hull classification rates for the 40 SCOP groups, where the natural vectors and the PseAAC vectors are partitioned into 10 dimensions. N1 refers to the first 10 dimensions of the natural vector, which are the numbers for amino acids A,R,N,D,C,Q,E,G,H,I; N2 refers to the second 10 dimensions of the natural vector, which are the numbers for amino acids L,K,M,F,P,S,T,W,Y,V. The other labels are similarly defined.

**Table 21:** The classification results for the 40 SCOP groups by the SVM method.

Feature methods	Classification rates by structural classes (%)			
	All α	All β	α + β	α/β
**NV**	100	64.89	54.26	76.32
**APF**	100	73.40	46.81	73.68
**PseAAC**	100	75.53	73.40	82.46
**NV, APF**	100	74.47	84.04	92.98
**NV, PseAAC**	100	82.98	94.68	90.35
**APF, PseAAC**	100	81.91	81.91	84.21
**NV, APF, PseAAC**	89.42	85.11	94.68	91.23
**PSSM**	100	100	100	100
**Average Rates**	98.68	79.79	78.72	86.40

This table shows the classification rates for the 40 SCOP groups by the SVM method.

**Table 22:** The classification results for the 40 SCOP groups by the random forest method.

Feature methods	Classification rates by structural classes (%)			
	All α	All β	α + β	α/β
**NV**	96.15	79.79	81.91	96.49
**APF**	92.31	74.47	68.09	96.49
**PseAAC**	95.19	77.66	89.36	98.25
**NV, APF**	98.08	80.85	81.91	96.49
**NV, PseAAC**	98.08	80.85	91.49	98.25
**APF, PseAAC**	97.12	79.79	92.55	99.12
**NV, APF, PseAAC**	99.04	82.98	91.49	97.37
**PSSM**	87.50	100	98.18	100
**Average Rates**	95.43	82.05	86.87	97.81

This table shows the classification rates for the 24 SCOP groups by the random forest method.

The classification results of the convex hull method are shown in Table 20. The convex hull classification rates attain overall higher classification rates than the multi-class MSE method, where the natural vector feature achieves the higher classification rates than the other methods. The PseAAC also presents good classification results, while the averaged property factor feature again presents the lowest classification rates. The average convex hull classification rates for the four structural classes are 95.43%, 82.05%, 86.87%, 97.81%. These convex hull results suggest that the four structural classes are separable by convex hulls in terms of the sequence features, and the convex hull method is more efficient in the structural identification than the multi-class MSE method.

In Tables 21 and 22, the SVM and the random forest methods also present good classification of the four structural classes in feature spaces. In Table 21, the SVM achieves the average classification rates of 98.68%, 79.79%, 78.72%, 86.40% for the four structural classes. In Table 22, the random forest method attains 95.43%, 82.05%, 86.87%, and 97.81% for the four structural classes. In this analysis the different structural classes are well separated in terms of the different feature methods, where the natural vector method outperforms the other feature methods in the structural classification.

#### SCOP III: 48 SCOP groups

The SCOP III dataset is composed of 48 SCOP groups. The protein ID and feature vectors of the 48 SCOP groups are given in the Supporting Information S11 and S12 Datasets. The MSE and convex hull classification results for the 40 SCOP groups are shown in Tables 23–26. In Table 23, the average MSE classification rates are 66.765, 56.00%, 59.68%, 65.22%. The four structural classes are identifiable by the natural vectors and the PseAAC. The averaged property factor method attains the lowest classification rates for most of the structural classes which cannot separate the four structural classes. However, when different types of features are combined, the classification rates are overall improved in comparison to their individual features.

**Table 23:** The classification results for the 48 SCOP groups by the multi-class MSE method.

Feature methods	Classification rates by structural classes (%)			
	All α	All β	α + β	α/β
**NV**	61.54	50.18	45.31	68.92
**APF**	48.77	57.22	45.31	63.18
**PseAAC**	66.61	61.44	48.08	57.88
**NV, APF**	73.16	60.04	70.05	69.81
**NV, PseAAC**	78.56	65.14	71.27	73.49
**APF, PseAAC**	67.10	63.20	54.84	59.06
**NV, APF, PseAAC**	77.91	67.08	72.20	75.26
**PSSM**	60.39	55.69	70.36	54.19
**Average Rates**	66.76	56.00	59.68	65.22

This table shows the multi-class MSE classification rates for the 48 SCOP groups with different feature combinations.

**Table 24:** The classification results for the 48 SCOP groups by the convex hull method.

Feature methods		Classification rates by structural classes (%)			
		All α	All β	α + β	α/β
**NV**	**N1**	80.03	87.32	84.79	86.45
	**N2**	85.11	89.08	84.64	82.33
	**Mu1**	63.01	65.85	68.20	38.14
	**Mu2**	68.41	61.97	63.90	58.32
	**D1**	54.99	54.40	64.98	30.04
	**D2**	57.94	54.58	61.60	37.11
**APF**		71.36	77.29	62.21	49.48
**PseAAC1**		70.54	77.99	70.20	57.58
**PseAAC2**		68.58	81.87	66.05	55.38
**PSSM**		80.31	99.74	92.53	97.20
**Average Rates**		70.03	75.01	71.91	59.20

This table shows the convex hull classification rates for the 48 SCOP groups, where the natural vectors and the PseAAC vectors are partitioned into 10 dimensions. N1 refers to the first 10 dimensions of the natural vector, which are the numbers for amino acids A,R,N,D,C,Q,E,G,H,I; N2 refers to the second 10 dimensions of the natural vector, which are the numbers for amino acids L,K,M,F,P,S,T,W,Y,V. The other labels are analogously defined.

**Table 25:** The classification results for the 48 SCOP groups by the SVM method.

Feature methods	Classification rates by structural classes (%)			
	All α	All β	α + β	α/β
**NV**	100	64.89	54.26	76.32
**APF**	100	73.40	46.81	73.68
**PseAAC**	100	75.53	73.40	82.46
**NV, APF**	100	74.47	84.04	92.98
**NV, PseAAC**	100	82.98	94.68	90.35
**APF, PseAAC**	100	81.91	81.91	84.21
**NV, APF, PseAAC**	100	97.78	81.67	97.50
**PSSM**	100	96.25	100	100
**Average Rates**	100	81.37	77.10	87.19

This table shows the classification rates for the 48 SCOP groups by the SVM method.

**Table 26:** The classification results for the 48 SCOP groups by the random forest method.

Feature methods	Classification rates by structural classes (%)			
	All α	All β	α + β	α/β
**NV**	84.45	77.46	86.02	91.90
**APF**	80.20	77.11	85.25	91.16
**PseAAC**	90.34	84.33	93.09	96.61
**NV, APF**	89.03	82.39	89.09	95.29
**NV, PseAAC**	92.14	86.44	94.16	97.79
**APF, PseAAC**	93.78	89.08	95.70	99.41
**NV, APF, PseAAC**	91.98	91.20	92.93	97.79
**PSSM**	98.86	100	89.16	88.12
**Average Rates**	90.10	86.00	90.68	94.76

This table shows the classification rates for the 48 SCOP groups by the random forest method.

In Table 24, the convex hull classification results suggest that the four structural classes are separable by the N features of the natural vectors and the PseAAC features. The average convex hull classification rates for the four structural classes are 70.03%, 75.01%, 71.91%, and 59.20%. Note that the convex hull classification rates are overall higher than the MSE results, which implies that the structural classes are better separated by the convex hulls than by the MSE hyper-planes. Moreover, the N features of the natural vector i.e. the amino acid composition the protein sequences present the higher classification results than other features, but the μ and D features of the natural vectors present the lower classification rates than all the other features. These results demonstrate that the importance of amino acid composition in the structural identification.

Tables 25 and 26 show the classification rates of the four structural classes by using the SVM and random forest methods. The SVM (Table 25) achieves the average classification rates of 100%, 81.37%, 77.10%, 87.19% for the four structural classes. In the SVM analysis, the natural vector presents slightly higher classification rates than the averaged property factor features. Similar situation happens for the random forest classification analysis. In Table 26, the random forest method achieves the average classification rates of 90.10%, 86.00%, 90.68%, 94.76% for the four structural classes.

#### SCOP IV: SCOP data with sequence similarity below 30%

In this section, we analyze all SCOP data in the PDB database with sequence similarity below 30%. We downloaded the PDB data of proteins obtained by X-Ray experiments and sequence similarity below 30%. The protein ID and feature vectors of the SCOP data with low sequence similarity are given in the Supporting Information S13 and S14 Datasets. The data information is shown in Table 14. We carry out the natural vector and the averaged property factor feature analysis and the multi-class MSE and the convex hull structural separation studies on this dataset, and compare the analysis with the PSSM feature analysis and the SVM and random forest classification methods. The results are shown in Tables 27–30.

**Table 27:** The classification results for the SCOP data with sequence similarity below 30% by the multi-class MSE method.

Feature methods	Classification rates by structural classes (%)			
	All α	All β	α + β	α/β
**NV**	61.15	52.82	51.84	52.08
**APF**	63.85	58.74	42.85	31.21
**PseAAC**	64.27	58.74	52.58	40.00
**NV, APF**	66.77	56.80	52.36	54.70
**NV, PseAAC**	68.85	58.35	50.96	55.70
**APF, PseAAC**	59.90	50.00	51.03	48.46
**NV, APF, PseAAC**	63.54	52.33	50.37	51.41
**PSSM**	43.54	41.07	26.25	31.21
**Average Rates**	61.48	53.61	47.28	45.60

This table shows the MSE classification rates for the SCOP data with sequence similarity below 30%.

**Table 28:** The classification results for the SCOP data with sequence similarity below 30% by the convex hull method.

Feature methods		Classification rates by structural classes (%)			
		All α	All β	α + β	α/β
**NV**	**N1**	76.56	69.61	58.19	56.51
	**N2**	78.75	69.13	63.42	64.50
	**Mu1**	60.83	60.78	59.66	55.64
	**Mu2**	66.98	52.62	61.21	64.03
	**D1**	69.48	66.21	63.86	66.58
	**D2**	71.67	63.40	66.81	71.14
**APF**		62.29	53.79	42.26	18.59
**PseAAC1**		60.21	51.55	43.73	19.13
**PseAAC2**		67.29	56.50	50.22	20.60
**PSSM**		67.50	60.00	57.52	32.48
**Average Rates**		68.16	60.36	56.69	46.92

This table shows the convex hull classification rates for the SCOP data with sequence similarity below 30%, where the natural vectors and PseAAC vectors are partitioned into 10 dimensions. N1 refers to the first 10 dimensions of the natural vector, which are the numbers for amino acids A,R,N,D,C,Q,E,G,H,I; N2 refers to the second 10 dimensions of the natural vector, which are the numbers for amino acids L,K,M,F,P,S,T,W,Y,V. The other labels are similarly defined.

**Table 29:** The classification results for the SCOP data with sequence similarity below 30% by the SVM method.

Feature methods	Classification rates by structural classes (%)			
	All α	All β	α + β	α/β
**NV**	100	26.89	57.67	96.85
**APF**	100	40.87	15.86	79.46
**PseAAC**	100	18.35	24.56	96.17
**NV, APF**	100	24.56	21.98	93.09
**NV, PseAAC**	100	28.84	25.44	97.72
**APF, PseAAC**	100	21.65	20.21	98.32
**NV, APF, PseAAC**	41.56	59.61	61.06	99.46
**PSSM**	47.50	83.98	45.28	99.93
**Average Rates**	86.13	38.09	34.01	95.13

This table shows the classification rates for the SCOP data with sequence similarity below 30% by the SVM method.

**Table 30:** The classification results for the SCOP data with low similarity by the random forest method.

Feature methods	Classification rates by structural classes (%)			
	All α	All β	α + β	α/β
**NV**	53.65	55.15	99.93	78.72
**APF**	54.27	59.61	98.53	47.45
**PseAAC**	50.83	56.60	99.93	52.01
**NV, APF**	60.21	60.68	100	79.26
**NV, PseAAC**	50.52	57.48	99.93	59.66
**APF, PseAAC**	53.65	57.86	100	51.07
**NV, APF, PseAAC**	62.81	64.37	100	79.66
**PSSM**	64.38	76.31	73.60	99.80
**Average Rates**	56.29	61.01	96.49	68.45

This table shows the classification rates for the SCOP data with sequence similarity below 30% by the random forest method.

In Table 27, the multi-class MSE method achieves the average classification rates of 61.48%, 53.61%, 47.28%, and 45.60% for the four structural classes. From this table, we can see that the four structural classes are separable in the natural vector feature space, and the natural vector feature presents overall higher classification rates than the averaged property factor method. The augmented features i.e. the combination of different features present higher classification rates than the individual feature method. The PSSM feature presents the lowest classification rates in this analysis.

In Table 28, the convex hull classification method presents good classification for the four structural classes. The average classification rates by the convex hull method are 68.16%, 60.36%, 56.69%, 46.92%. The classification rates of the natural vector features are higher than the classification rates of the averaged property factor features. The SVM classification (Table 29) shows lower classification rates than the multi-class and the convex hull classification methods, the average classification rates for the four structural classes are 86.13%, 38.09%, 34.01%, 95.13%. The SVM does not perform well in this analysis. The random forest well separates the four structural classes (Table 30), the average classification rates for the four structural classes are 56.29%, 61.01%, 96.49%, and 68.45%. The natural vector method presents higher classification rates than the averaged property factor method.

## Discussion

In this paper, we use protein sequence features to study the structural separation of proteins. We use two typical protein sequence features, namely the natural vector method and the averaged property factor method, to extract protein sequence features. The natural vector focuses on the composition and sequence arrangements of amino acids, while the averaged property factor focuses on the physical properties of amino acids. We compare the two feature methods with the PseAAC and the PSSM features. These feature methods map protein sequences into high-dimensional real vectors, where we use the multi-class MSE and the convex hull methods to classify these feature vectors into separate regions. We aim to inspect whether the different secondary structural classes are separable in terms of the sequence features, and also to check which kind of sequence features better influence the structures. The classification analysis is compared with traditional methods such as the SVM and the random forest methods.

We use three CATH datasets and four SCOP datasets to demonstrate the analysis. We found that the different structural classes of CATH and SCOP are separable by hyper-planes and convex hulls in the sequence feature spaces, the natural vector method outperforms the other feature methods in nearly all structural classifications. As compared among the different classification methods, the multi-class MSE, the convex hull method, and the random forest method have good performances in the structural classification. The SVM presents good classifications in most cases, but it may have no convergence in the computation for some large datasets due to the high dimensions of the feature vectors, and the classification rates of the SVM are sometimes lower than the results of the other methods. The convex hull method presents the best classifications to the structural classes than the other methods.

In the feature analysis, we compared the natural vector and the averaged property factor methods with the PseAAC and the PSSM feature methods. The natural vector is claimed to have one-to-one correspondence with the protein sequence [4], it is composed of three major parts that representing the compositions, positions and the sequence arrangements of the common 20 types of amino acids. The averaged property factor method [18] focuses on the 10 physical properties of amino acids. The PseAAC method [21–23] presents the amino acid compositions of protein sequences which is often use by machine learning classification methods [31–33]. The PSSM is a position specific scoring method that scores the local alignment profile of protein sequences [24–25]. We use these four feature methods to present the protein sequences, the natural vector method and the PseAAC method often present better performances in the structural classification than the averaged property factor method. Particularly, in the low similarity data analysis, the natural vector method presents apparent superiority than the averaged property factor method in the convex hull classification. These may imply that the amino acid composition and their sequence arrangements presented by the natural vectors may have better inference to the protein structures than the averaged physical properties of the amino acids. Note that the PSSM is different from the natural vector in extracting the sequence features. The natural vector counts both the composition and the sequence arrangements of amino acids in protein sequences, it computes the average distance and moments of each type of amino acids to the origin (i.e. the first amino acid of the sequence). The amino acid composition, the average distance and the moments together is a hallmark of each individual sequence, and does not relying on the alignment of other sequences. The PSSM scores the alignment of the sequence and these scores depend on the alignment of the sequence to other sequences. In most cases, the PSSM presents higher classification rates than the averaged property factor method, as the natural vector method does.

In most of the cases, the combination of different features presents better classification results than the individual features. Usually, the complete combination of all three methods presents the highest classification results than any other combinations. The classification rates are apparently improved when including the natural vectors and the PseAAC features, which imply that the amino acid composition and their sequence arrangements may have great influence to the structures.

In the classification study, we use multi-class MSE and convex hull methods to study the separation of feature spaces, which are compared with the analysis of the SVM and the random forest method. Results demonstrate that the different structural classes of CATH and SCOP are separable by using the multi-class MSE and the convex hull methods in terms of the natural vector features. The natural vector outperforms the averaged property factor method in nearly all classifications.

The convex hull classification results are comparatively higher than the multi-class MSE results in nearly all simulation studies. This implies that the convex hulls present better separation for the feature points than the MSE hyper-planes. The ‘exclusively in hull’ of the convex hull method has good advantages over the regional ‘cuts’ by the MSE hyper-planes in the structural separation of feature spaces. Both the classification methods are popular classifiers, but they present the classification in different manners. The MSE classifier is often used in machine learning classifications, which cuts real spaces into disjoint regions [19]. The convex hull method is used for taxonomy or evolutionary classification for genes or proteins [20], where the feature points of different genetic families or taxa are enclosed in different convex hulls. Although, the convex hull method presents better classification rates in the structural separation, both classifiers support the same results that the different structural classes are separable in the natural vector spaces, which admits better separation of the structural classes than the other feature methods.

The classification rate defined with the MSE and convex hull methods is used to quantify the separation quality for the different structural classes. It measures the ‘exclusiveness’ of feature points in the region of each structural class. Note that different sequences may correspond to similar structures, while similar sequences may also correspond to different structures. Therefore, the structural separation in the feature spaces does not mean the exact classification of the structures, but is a general division of the feature spaces. From this study, we see that the feature points of different structural classes occupy different regions in the feature spaces, which can be separated by the hyper-planes and convex hulls. The overall results address the important connections between the protein sequences (the amino acid composition and sequence arrangements) and their structures.

## Conclusion

In this study, we use the multi-class MSE and the convex hull methods to separate the protein structural classes in the protein sequence feature spaces. We found that the different structural classes of CATH and SCOP are separable by hyper-planes and convex hulls in terms of the natural vector features. The natural vector method outperforms the averaged property factor method in the structural separation, and the convex hull method outperforms the multi-class MSE method in the structural separation of feature spaces. The results may imply that the amino acid composition and their sequence arrangements presented by the natural vectors may have better indications to the structures than the averaged physical properties of amino acids.

## Supporting information

S1 Table The 10 property factors of the 20 amino acids [29–30]. This table shows the 10 property factors of the 20 types of amino acids. The order of the 10 properties of amino acids are 1. Alpha-helix/bend preference; 2. Side-chain size; 3. Extended structure preference; 4. Hydrophobicity; 5. Double-bend preference; 6. Amino acid composition; 7. Flat extended preference; 8. Occurrence in region; 9. pk; 10. Surrounding hydrophobicity.
(DOCX)

S1 Dataset The data description and PDB IDs of the 30 CATH groups (CATH I). This is the data description and PDB IDs of the 30 CATH groups (CATH I).
(RAR)

S2 Dataset The feature vectors of the 30 CATH groups (CATH I). This is the data file for the feature vectors of the 30 CATH groups (CATH I).
(MAT)

S3 Dataset The data description and PDB IDs of the 40 CATH groups (CATH II). This is the data description and PDB IDs of the 40 CATH groups (CATH II).
(RAR)

S4 Dataset The feature vectors of the 40 CATH groups (CATH II). This is the data file for the feature vectors of the 40 CATH groups (CATH II).
(MAT)

S5 Dataset The PDB IDs of the CATH data with sequence similarity below 30% (CATH III). This is the PDB IDs of the CATH data with sequence similarity below 30% (CATH III).
(RAR)

S6 Dataset The feature vectors of the CATH data with sequence similarity below 30% (CATH III). This is the data file for the feature vectors of the CATH data with sequence similarity below 30% (CATH III).
(MAT)

S7 Dataset The data description and PDB IDs of the 24 SCOP groups (SCOP I). This is the data description and PDB IDs of the 24 SCOP groups (SCOP I).
(RAR)

S8 Dataset The feature vectors of the 24 SCOP groups (SCOP I). This is the data file for the feature vectors of the 24 SCOP groups (SCOP I).
(MAT)

S9 Dataset The data description and PDB IDs of the 40 SCOP groups (SCOP II). This is the data description and PDB IDs of the 40 SCOP groups (SCOP II).
(RAR)

S10 Dataset The feature vectors of the 40 SCOP groups (SCOP II). This is the data file for the feature vectors of the 40 SCOP groups (SCOP II).
(MAT)

S11 Dataset The data description and PDB IDs of the 48 SCOP groups (SCOP III). This is the data description and PDB IDs of the 48 SCOP groups (SCOP III).
(RAR)

S12 Dataset The feature vectors of the 48 SCOP groups (SCOP III). This is the data file for the feature vectors of the 48 SCOP groups (SCOP III).
(MAT)

S13 Dataset The PDB IDs of the SCOP data with sequence similarity below 30% (SCOP IV). This is the PDB IDs of the SCOP data with sequence similarity below 30% (SCOP IV).
(RAR)

S14 Dataset The feature vectors of the SCOP data with sequence similarity below 30% (SCOP IV). This is the data file for the feature vectors of the SCOP data with sequence similarity below 30% (SCOP IV).
(MAT)
