# Supplementary material for: A study on separation of the protein structural types in amino acid sequence feature spaces
Source: PLoS One. 2019 Dec 23;14(12):e0226768. doi: 10.1371/journal.pone.0226768 (PMC6927603; doi:10.1371/journal.pone.0226768)
Supplement: S1 Table — This table shows the 10 property factors of the 20 types of amino acids. The order of the 10 properties of amino acids are 1. Alpha-helix/bend preference; 2. Side-chain size; 3. Extended structure preference; 4. Hydrophobicity; 5. Double-bend preference; 6. Amino acid composition; 7. Flat extended preference; 8. Occurrence in region; 9. pk; 10. Surrounding hydrophobicity. (DOCX) [file pone.0226768.s001.docx]

**S1 Table. The 10 property factors of the 20 amino acids [29-30]**.

| **Amino acids** | **Abbreviation** | **1** | **2** | **3** | **4** | **5** | **6** | **7** | **8** | **9** | **10** |
| --- | --- | --- | --- | --- | --- | --- | --- | --- | --- | --- | --- |
| **ALA** | **A** | -1.56 | -1.67 | -0.97 | -0.27 | -0.93 | -0.78 | -0.20 | -0.08 | 0.21 | -0.48 |
| **ARG** | **R** | 0.22 | 1.27 | 1.37 | 1.87 | -1.70 | 0.46 | 0.92 | -0.39 | 0.23 | 0.93 |
| **ASN** | **N** | 1.14 | -0.07 | -0.12 | 0.81 | 0.18 | 0.37 | -0.09 | 1.23 | 1.10 | -1.73 |
| **ASP** | **D** | 0.58 | -0.22 | -1.58 | 0.81 | -0.92 | 0.15 | -1.52 | 0.47 | 0.76 | 0.70 |
| **CYS** | **C** | 0.12 | -0.89 | 0.45 | -1.05 | -0.71 | 2.41 | 1.52 | -0.69 | 1.13 | 1.10 |
| **GLN** | **Q** | -0.47 | 0.24 | 0.07 | 1.10 | 1.10 | 0.59 | 0.84 | -0.71 | -0.03 | -2.33 |
| **GLU** | **E** | -1.45 | 0.19 | -1.61 | 1.17 | -1.31 | 0.40 | 0.04 | 0.38 | -0.35 | -0.12 |
| **GLY** | **G** | 1.46 | -1.96 | -0.23 | -0.16 | 0.10 | -0.11 | 1.32 | 2.36 | -1.66 | 0.46 |
| **HIS** | **H** | -0.41 | 0.52 | -0.28 | 0.28 | 1.61 | 1.01 | -1.85 | 0.47 | 1.13 | 1.63 |
| **ILE** | **I** | -0.73 | -0.16 | 1.79 | -0.77 | -0.54 | 0.03 | -0.83 | 0.51 | 0.66 | -1.78 |
| **LEU** | **L** | -1.04 | 0.00 | -0.24 | -1.10 | -0.55 | -2.05 | 0.96 | -0.76 | 0.45 | 0.93 |
| **LYS** | **K** | -0.34 | 0.82 | -0.23 | 1.70 | 1.54 | -1.62 | 1.15 | -0.08 | -0.48 | 0.60 |
| **MET** | **M** | -1.40 | 0.18 | -0.42 | -0.73 | 2.00 | 1.52 | 0.26 | 0.11 | -1.27 | 0.27 |
| **PHE** | **F** | -0.21 | 0.98 | -0.36 | -1.43 | 0.22 | -0.81 | 0.67 | 1.10 | 1.71 | -0.44 |
| **PRO** | **P** | 2.06 | -0.33 | -1.15 | -0.75 | 0.88 | -0.45 | 0.30 | -2.30 | 0.74 | -0.28 |
| **SER** | **S** | 0.81 | -1.08 | 0.16 | 0.42 | -0.21 | -0.43 | -1.89 | -1.15 | -0.97 | -0.23 |
| **THR** | **T** | 0.26 | -0.70 | 1.21 | 0.63 | -0.10 | 0.21 | 0.24 | -1.15 | -0.56 | 0.19 |
| **TRP** | **W** | 0.30 | 2.10 | -0.72 | -1.57 | -1.16 | 0.57 | -0.48 | -0.40 | -2.30 | -0.60 |
| **TYR** | **Y** | 1.38 | 1.48 | 0.80 | -0.56 | -0.00 | -0.68 | -0.31 | 1.03 | -0.05 | 0.53 |
| **VAL** | **V** | -0.74 | -0.71 | 2.04 | -0.40 | 0.50 | -0.81 | -1.07 | 0.06 | -0.46 | 0.65 |

This table shows the 10 property factors of the 20 types of amino acids [29-30]. The order of the 10 properties of amino acids are 1. Alpha-helix/bend preference; 2. Side-chain size; 3. Extended structure preference; 4. Hydrophobicity; 5. Double-bend preference; 6. Amino acid composition; 7. Flat extended preference; 8. Occurrence in region; 9. pk; 10. Surrounding hydrophobicity.
